# Transcriptional Responses and GCMS Analysis for the Biosynthesis of Pyrethrins and Volatile Terpenes in *Tanacetum coccineum*

**DOI:** 10.3390/ijms222313005

**Published:** 2021-11-30

**Authors:** Tuo Zeng, Jia-Wen Li, Li Zhou, Zhi-Zhuo Xu, Jin-Jin Li, Hao Hu, Jing Luo, Ri-Ru Zheng, Yuan-Yuan Wang, Cai-Yun Wang

**Affiliations:** 1 A Key Laboratory for Biology of Horticultural Plants, Ministry of Education, College of Horticulture & Forestry Sciences, Huazhong Agricultural University, Wuhan 430070, China; zengtuo@gznu.edu.cn (T.Z.); lijiawen@webmail.hzau.edu.cn (J.-W.L.); zhouli@webmail.hzau.edu.cn (L.Z.); xzz1122@webmail.hzau.edu.cn (Z.-Z.X.); jinjinsweet@mail.hzau.edu.cn (J.-J.L.); haohu@mail.hzau.edu.cn (H.H.); ljcau@mail.hzau.edu.cn (J.L.); rrzheng@mail.hzau.edu.cn (R.-R.Z.); wangyy@mail.hzau.edu.cn (Y.-Y.W.); 2 School of Life Sciences, Guizhou Normal University, Guiyang 550025, China

**Keywords:** *Tanacetum coccineum*, pyrethrins, transcriptome, gene expression, GCMS analysis

## Abstract

Natural pyrethrins have been widely used as natural pesticides due to their low mammalian toxicity and environmental friendliness. Previous studies have mainly focused on *Tanacetum* *cinerariifolium*, which contains high levels of pyrethrins and volatile terpenes that play significant roles in plant defense and pollination. However, there is little information on *T. coccineum* due to its lower pyrethrin content and low commercial value. In this study, we measured the transcriptome and metabolites of the leaves (L), flower buds (S1), and fully blossomed flowers (S4) of *T. coccineum*. The results show that the expression of pyrethrins and precursor terpene backbone genes was low in the leaves, and then rapidly increased in the S1 stage before decreasing again in the S4 stage. The results also show that pyrethrins primarily accumulated at the S4 stage. However, the content of volatile terpenes was consistently low. This perhaps suggests that, despite *T. coccineum* and *T. cinerariifolium* having similar gene expression patterns and accumulation of pyrethrins, *T. coccineum* attracts pollinators via its large and colorful flowers rather than via inefficient and metabolically expensive volatile terpenes, as in *T. cinerariifolium.* This is the first instance of de novo transcriptome sequencing reported for *T. coccineum*. The present results could provide insights into pyrethrin biosynthetic pathways and will be helpful for further understanding how plants balance the cost–benefit relationship between plant defense and pollination.

## 1. Introduction

The *Tanacetum* genus belongs to the Asteraceae family and contains more than 160 accepted species (http://www.theplantlist.org, accessed on 18 July 2021). Most of these species possess high numbers of secondary metabolites with various biological functions, such as antibacterial, antioxidant, and herbicidal effects [1,2]. However, only a few species of this genus have efficient insecticidal effects, such as *T. cinerariifolium* and *T. coccineum*, which produce pyrethrins, highly effective natural insecticides. Pyrethrins are a mixture of six homologous esters, comprising cinerin I, jasmolin I, pyrethrin I (class I), cinerin II, jasmolin II, and pyrethrin II (class II) [3]. They are formed via the esterification of an acid moiety (chrysanthemic acid or pyrethric acid) and alcohol moiety (pyrethrolone, cinerolone, or jasmolone) [4]. Studies on *T. cinerariifolium* have shown that the alcohol moiety is derived from jasmonic acid (JA) biosynthetic pathways, and the jasmone is converted to jasmolone by the jasmolone hydroxylase (JMH) enzyme [5]. Then, jasmolone is converted to pyrethrolone by PYS, also known as CYP82Q3, belonging to the CYP82 gene family [6]. However, the enzyme for the conversion of jasmolone to cinerolone has not yet been reported. For the acid moiety, the first step is catalyzed by chrysanthemyl diphosphate synthase (CDS), which catalyzes the condensation of two dimethylallyl diphosphate (DMAPP) molecules to chrysanthemol [7,8]; this enzyme contains a plastidial signal peptide that binds the DMAPP derived from the methylerythritol phosphate (MEP) pathway [9]. Subsequently, chrysanthemol is oxidized into chrysanthemal and then into chrysanthemic acid by alcohol dehydrogenase (ADH) and aldehyde dehydrogenase (ALDH) [10]. The enzymes chrysanthemol 10-hydroxylase (CHH) and 10-methyltransferase (MT) process the chrysanthemic acid, turning it into pyrethric acid [11]. Finally, GDSL lipase-like protein (GLIP) forms the final pyrethrin molecule by esterifying the acid moiety and the alcohol moiety [12]. Because natural pyrethrins are minimally toxic to mammals and are friendly to the environment, pyrethrum species, especially *T. cinerariifolium*, are among the most commercially viable insecticidal plants [13].

Pyrethrins are mainly biosynthesized in flowers, and almost 94% of total pyrethrins are contained in the flower head [14]. Although seven developmental stages (S1–S7) exist, characterized by flower development [15], the genes for the biosynthesis of pyrethrins are mainly expressed during the S1 stage (flower bud). However, the pyrethrin content per unit of dry weight is optimal in the S4 stage (full-blossom flowers). In addition to pyrethrins, there are many other terpene compounds in *T. cinerariifolium*, the most important of which are (E)-beta-farnesene (EβF) and germacrene D (GD), derived from the mevalonate (MVA) pathway. Both are mainly synthesized at the flowering stage and account for almost 70% of the total terpenes at the bud stage—the precursor IPP and its isomer DMAPP are processed to form farnesyl diphosphate (FPP) by FPP synthase (FPS). FPP is converted to GD by the germacrene D synthase enzyme, or converted to EβF by the (E)-beta-farnesene synthase enzyme, respectively. EβF is the main component of natural aphid alarm pheromone [16], and lady beetles can capture this signal to search for and locate potential food sources [17]. EβF can also attract female hoverflies to spawn upon the plants [18]. Studies have shown that EβF can attract lady beetles in *T. cinerariifolium*. It can also repel aphids or achieve defense by synergistically enhancing the repellent effect of pyrethrins on mosquitoes [17,19]. GD, as an insect pheromone mimic, may contribute to overall insect defense [20].

The functions of these terpenes extend far beyond plant defense. *T. cinerariifolium* is a highly heterozygous, self-sterile species [21], so it needs insects to pollinate. Plants often attract pollinating insects through flower color and fragrance [22]. *T. cinerariifolium* may not be a plant favored by pollination insects due to its lack of typical floral fragrance and flower color. However, field observations have shown that *T. cinerariifolium* can attract a large number of hoverflies and that lady beetles, relatively rare bees, and rare butterflies pollinate upon the flower heads [17]. The lady beetle and the hoverfly can provide considerable pollination services [23]. EβF is the most abundant substance in the volatiles at the bud stage. After that, as the flowers are flowering, the tubular flowers in the middle of the flower heads gradually open. The release of GD increases rapidly, and GD becomes the most abundant volatile in the blooming period. EβF and GD may play important roles in attracting pollinators [24]. This result fully shows that the terpenes and pyrethrins play essential functions in plant defense and pollination in *T. cinerariifolium*.

*T. coccineum* is a species related to *T. cinerariifolium* in the *Tanacetum* genera. It is native to the Caucasus region between the Caspian Sea and the Black Sea [25]. Its dried flower powder is usually called “Persian insect powder” and contains a considerable amount of pyrethrins. It was used to delouse children in ancient Persia [26]. Unlike that of *T. cinerariifolium*, the flower color of *T. coccineum* is highly variable, varying from white to pink and red to maroon [27], so it is widely used in ornamental gardening and as a horticultural protection plant. Observations of *T. coccineum* show that frequent butterfly visits occur on its flower heads. Therefore, the plant has significant economic value and is suitable for studying the relationship between plant defense and pollination. However, current reports on *T. coccineum* are scarce. Only the anatomical and histochemical characteristics and the chloroplast genome have been studied [28,29]. This study determined the pyrethrins and volatile metabolites for typical leaves and different flowering stages of *T. coccineum* by transcriptomic analysis and GCMS. It provides valuable molecular information on the biosynthesis of pyrethrins in *T. coccineum* and inspiration to further explore the relationship between plant defense and pollination in pyrethrums. It will also help the future genetic improvement of *T. coccineum*.

## 2. Results

### 2.1. Transcriptome Sequencing and de novo Assembly of T. coccineum

RNA-Seq of *T. coccineum* was performed using the Illumina HiSeq™ 4000 sequencing platform with three samples—flash leaves (L), flower buds (S1), and fully blossomed flowers (S4)—with a total of nine sequencing libraries. The results in Table S1 show that at least 43,845,892 raw reads were obtained in each library, and at least 42,370,462 clean reads and 6.36 G clean bases were obtained for each library after removing the sequencing linker, low-quality reads, and reads with N ratios greater than 10%. The percentages of clean reads in all nine libraries were over 94%, the percentage with Q30 was at least 93.15%, and the GC percentage was about 42.6%. These results indicate that the sequencing was of sufficient quality. 

**Figure 1:**
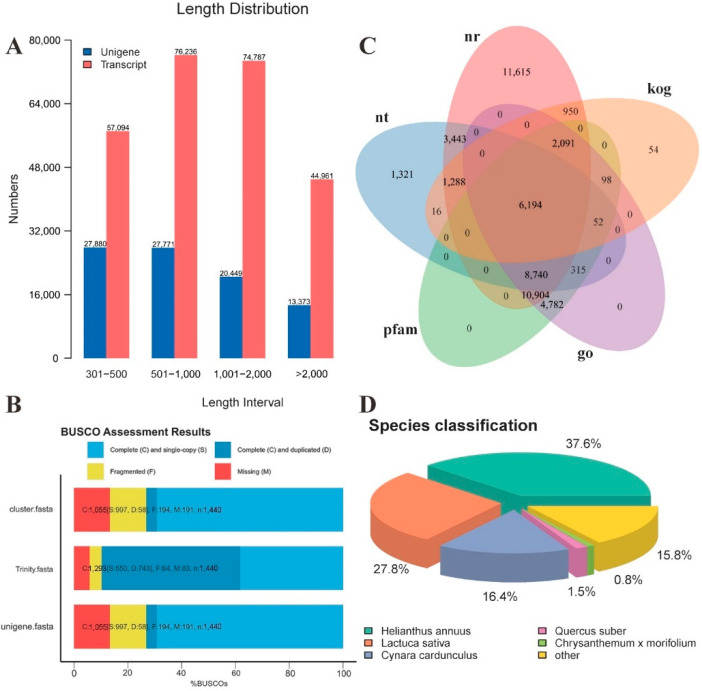
Transcriptome assembly and gene functional annotation. (**A**) Length distributions of assembled transcripts and unigenes; (**B**) BUSCO assessment results; (**C**) total number of functional annotations in Nt, Nr, KOG, GO, and Pfam databases; (**D**) species distribution of the BLASTX result against the Nr database.

We used the Trinity software to assemble the clean reads of *T. coccineum* in nine libraries. This also generated 253,078 transcripts and 89,473 unigenes. The average read length and N50 length were 1245 and 1732 bp in the transcripts and 1107 and 1637 in the unigenes, respectively. The length distributions of the transcripts and unigenes are shown in (Figure 1A). The results indicate that the numbers of unigene fragments with lengths of 301–500, 501–1000, 1001–2000, and greater than 2000 bp were 27,880 (31.16%), 27,771 (31.03%), 20,449 (22.85%), and 13,373 (14.95%), respectively. BUSCO analysis also showed that most genes could be mapped to the BUSCO (Figure 1B). These results show that the sequencing data were of sufficient quality and could be used in subsequent analyses.

### 2.2. Functional Annotation of Unigenes

All the assembled unigenes were blasted for functional annotation analysis against public databases, including Nr, Nt, PFAM, GO, KOG, KO, and SwissProt, using the BLASTx program. The annotation results for the first five databases are shown by the Venn diagram in Figure 1C. The results show that 45,225 unigenes (50.54%) could be annotated in the Nr database, 21,369 (23.88%) in the Nt database, 15,903 (17.77%) in the KO database, 33,456 (37.39%) in the SwissProt database, 33,176 (37.07%) in the PFAM database, 33,176 (37.07%) in the GO database, and 10,743 (12%) in the KOG database. Additionally, 5017 (5.6%) unigenes were annotated in each database, and 52,057 (58.18%), in at least one database.

The species statistics of the unigenes annotated by the Nr database were determined. The top five annotated species were Helianthus annuus, Lactuca sativa, Cynara cardunculus, Quercus suber, and Chrysanthemum × morifolium. These accounted for 37.6%, 27.8%, 16.4%, 1.5%, and 0.8% of the total number of Nr annotations, respectively (Figure 1D).

### 2.3. Differentially Expressed Gene (DEG) Analysis

**Figure 2:**
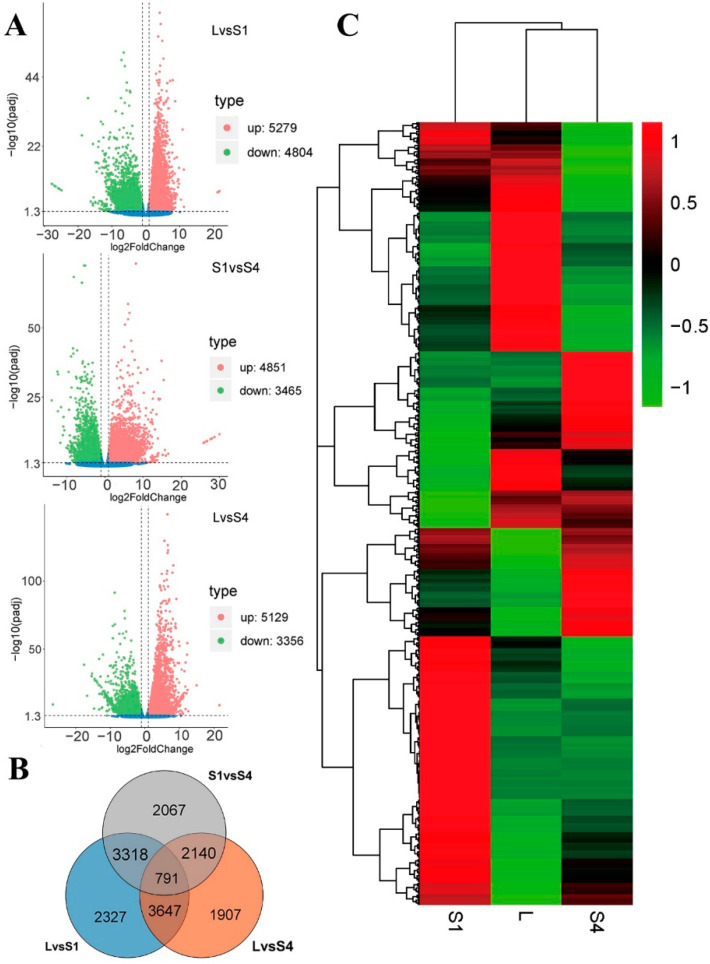
Analysis of DEGs in *T. coccineum*. (**A**) Volcano plots for LvsS1, LvsS4, and S1vsS4, from top to bottom; vertical dotted lines indicate a Log_2_ fold change of 1, horizontal dotted line indicates an adjusted p-value of 0.05 (**B**) Venn diagram showing the numbers of identified DEGs for LvsS1vsS4; (**C**) heat map of DEG clusters.

The PCA and Pearson correlation analysis of these samples showed consistency among each biological replicates (Figure S1). To identify DEGs among the samples, we compared them with each other and identified significant DEGs with Padj < 0.05 and |log_2_FoldChange| > 1. The results showed that the LvsS1 comparison group identified 10,083 DEGs, of which 5279 were up-regulated and 4804 were down-regulated. The LvsS4 comparison group identified 8485 DEGs, with 5129 up-regulated and 3356 down-regulated. The S1vsS4 comparison group revealed 8316 DEGs, with 4851 up-regulated and 3465 down-regulated (Figure 2A). There were 791 common DEG changes in the three comparative transcriptomes (Figure 2B). To visualize the DEGs of *T. coccineum*, we generated a heat map via hierarchical clustering (Figure 2C). The heat map showed that the up- and down-regulated DEGs compared with L in the S1 and S4 sample groups had either the same or different expression trends, indicating that *T. coccineum* with S1 and S4 stages had both common and unique gene expression patterns.

### 2.4. GO and KEGG Enrichment Analysis of DEGs

To further explore the possible functions of DEGs, GO enrichment analysis of the most significant 30 terms of DEGs was performed (Figure S2). The results show that the common and differentially expressed genes in the three comparison groups were mainly enriched in the metabolic process (3862, 3302, and 3253), catalytic activity (3417, 2942, and 2836), oxidoreductase activity (782, 734, and 663), and oxidation reduction process (743, 695, and 622). However, there were also individual differences, such as LvsS1 being mainly enriched in small molecule binding (1334), nucleotide binding (1297), nucleoside phosphate binding (1297), and other binding nodes. In LvsS4, there were mainly metabolic processes of single-organism metabolic process (1558), single organism biosynthetic process (468), and organic acid metabolic process (416). The differentially expressed genes in S1vsS4 were mainly enriched in nodes, such as transferase activity (1184), carbohydrate metabolic process (354), and nucleic acid binding transcription factor activity (232).

All the DEGs were mapped on the KEGG database to search for DEGs involved in metabolic or signal-transduction pathways. The 20 pathway items with the most significant enrichment are displayed in Figure S3. The KEGG pathways were co-enriched in the three comparison groups, which included starch and sucrose metabolism (93, 94, and 103) and amino sugar and nucleotide sugar metabolism (59, 58, and 72). The metabolism terms co-enriched by LvsS1 and LvsS4 were glyoxylate and dicarboxylate metabolism (68 and 64), carbon fixation in photosynthetic organisms (53 and 57), porphyrin and chlorophyll metabolism (46 and 50), photosynthesis (45 and 44), and terpenoid backbone biosynthesis (42 and 32). There were, however, differences among the comparison groups, such as for cysteine and methionine metabolism (49) and peroxisome (47) for LvsS1; plant hormone signal transduction (103) and glycine, serine, and threonine metabolism (47) for LvsS4; and ribosome (176), plant hormone signal transduction (96), and phenylpropanoid biosynthesis (81) for S1vsS4.

### 2.5. DEGs Involved in the Terpenoid Biosynthesis Pathway

**Figure 3:**
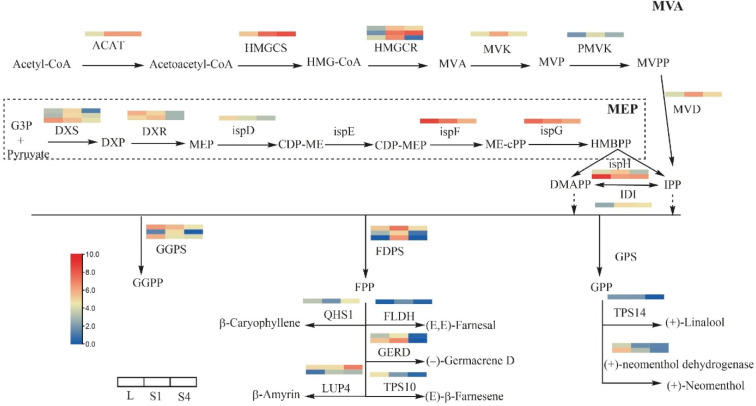
DEGs assigned to terpenoid biosynthesis pathway. The colors show the normalized expression scores from log_2_(FPKM+1) of DEGs. Abbreviations: DXS, 1-deoxy d-xylulose 5-phosphate synthase; DXR, 1-deoxy-D-xylulose-5-phosphate reductoisomerase; ISPD, 2-C-methyl-D-erythritol 4-phosphate cytidylyltransferase; ISPE, 4-diphosphocytidyl-2-C-methyl-D-erythritol kinase; ISPF, 2-C-methyl-D-erythritol 2,4-cyclodiphosphate synthase; ISPG, (E)-4-hydroxy-3-methylbut-2-enyl-diphosphate synthase; ISPH, 4-hydroxy-3-methylbut-2-en-1-yl diphosphate reductase; IDI, isopentenyl-diphosphate delta-isomerase; ACAT, acetyl-CoA C-acetyltransferase; HMGS, hydroxymethylglutaryl-CoA synthase; HMGR, 3-hydroxy-3-methylglutaryl-coenzyme A reductase; MVK, mevalonate kinase; PMVK, phosphomevalonate kinase; MVD, diphosphomevalonate decarboxylase; GPS, geranyl diphosphate synthase; TPS, farnesyl diphosphate synthase; GGPS, geranylgeranyl diphosphate synthase; LUP4, beta-amyrin synthase; GERD, (−)-germacrene D synthase; SQLE, squalene epoxidase.

Terpene precursors are derived from mevalonic acid (MVA) and methylerythritol phosphate (MEP) pathways, so we studied the DEGs for terpenes in *T. coccineum* (Figure 3). Based on DEG analysis and the gene annotation, these genes included HMGCR (3), DXS (3), DXR (2), ispH (2), GGPS (3), FDPS (3), GERD (2), LUP4 (2), (+)- neomenthol dehydrogenase (2), and others with one gene. The results show that most of the terpene-precursor synthesis genes were expressed at low levels in the L and S4 phases, while the S1 phase was the highest in terpene gene expression.

### 2.6. DEGs Involved in Pyrethrin Biosynthetic Pathways

**Figure 4:**
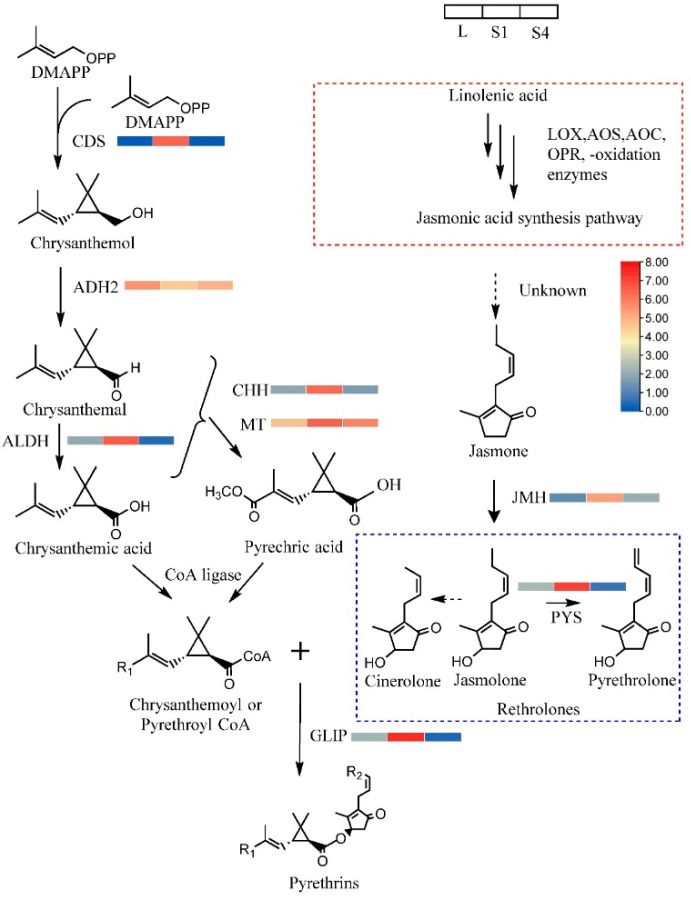
The biosynthesis of pyrethrin and gene expression. The color shows the normalized expression score from log_2_(FPKM+1) of DEGs. Solid arrows indicate known steps in the pathway, dotted arrows indicate steps not yet elucidated, red dotted box indicates the JAs synthesis pathway, blue dotted box indicates the rethrolones, a pyrethrin molecule incorporates one of three rethrolones. Abbreviations: CDS, chrysanthemol synthase; ADH2, alcohol dehydrogenase 2; ALDH, aldehyde dehydrogenase; CHH, chrysanthemol 10-hydroxylase; MT, 10-carboxychrysanthemic acid 10-methyltransferase; PYS, pyrethrolone synthase; JMH, jasmolone hydroxylase; GLIP, GDSL lipase-like protein.

Pyrethrins are efficient natural insecticides that are created by some species belonging to the Asteraceae family. The pyrethrin biosynthesis pathway in *T. cinerariifolium* has been clarified relatively well. However, no study has focused on the biosynthesis of pyrethrins in *T. coccineum*. The genes homologous with the genes that were clearly identified in *T. cinerariifolium*, such as ADH (MF497444.1), ALDH (MF497445.1), CDS (JX913536.1), CHH (KC441525.1), MT (MK139710.1), JMH (MG189934.1), GLIP (JN418994.1 ), and PYS (MG874680.1), were identified through BLASTn. Based on DEG analysis and sequence alignment, the pyrethrin synthesis genes were extremely conserved in the two species, and the primary gene for pyrethrin synthesis was minimally expressed in leaves, increased in the S1 stage, and decreased again in the S4 stage, with co-expression patterns (Figure 4) similar to the observations in *T. cinerariifolium*.

### 2.7. DEGs Involved in Anthocyanin Biosynthetic Pathways

**Figure 5:**
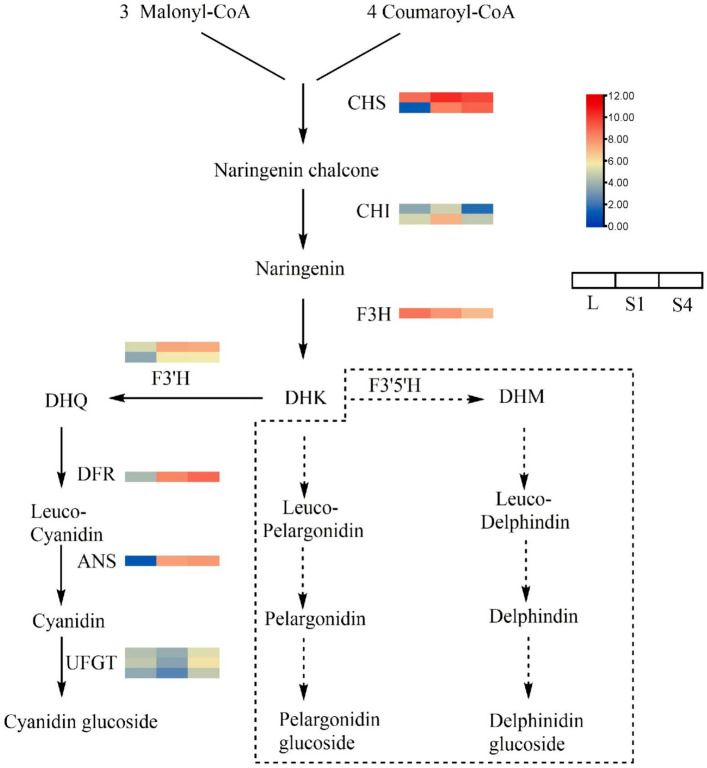
The biosynthesis of anthocyanin and gene expression. The color shows the normalized expression score from log_2_ (FPKM+1) of DEGs. Solid lines, pathways present; dotted box and dotted arrows, pathways absent. Abbreviations: chalcone synthase, CHS; chalcone isomerase, CHI; flavanone 3-hydroxylase, F3H; flavonoid 3’-hydroxylase, F3’H; dihydroflavonol 4-reductase, DFR; anthocyanidin synthase, ANS; dihydrokaempferol, DHK; dihydroquercetin, DHQ; dihydromyricetin, DHM; UDP-glucosyl transferase, UFGT.

*T. coccineum* flowers can blossom in various shades of red, pink, and orange as opposed to the white flower color of *T. cinerariifolium*. To investigate the genes related to the biosynthesis of anthocyanin, the DEG analysis combined the homology genes identified through BLAST with the genes identified in C× morifolium (Figure 5), such as chalcone synthase, CHS (DQ521272.1); chalcone isomerase, CHI (EU286277.1); flavanone 3-hydroxylase, F3H (U86837.1); flavonoid 3’-hydroxylase, F3’H (MF663713.1); dihydroflavonol 4-reductase, DFR (GU324979.1); and anthocyanidin synthase, ANS (EU810810.2) [30,31,32]. These genes included CHS (2), CHI (2), F3H (1), F3’H (2), DFR (1), ANS (1), and UDP-glucosyl transferase UFGT (3). The results show that the anthocyanin expression of *T. coccineum* is very similar to that of C× morifolium. It lacks the gene that can synthesize delphinidin, so it can not appear blue. Unlike the expression of genes involved in the biosynthesis of pyrethrins, which are mainly expressed in the S1 stage, early anthocyanin biosynthesis genes, such as CHS and CHI, are mainly highly expressed in the S1 stage and expressed less in other stages. By contrast, late anthocyanin biosynthesis genes DFR and ANS are expressed less in leaves but increase in the S1 stage, and are expressed at the highest level in the S4 stage.

### 2.8. qRT-PCR Validation of DEGs

**Figure 6:**
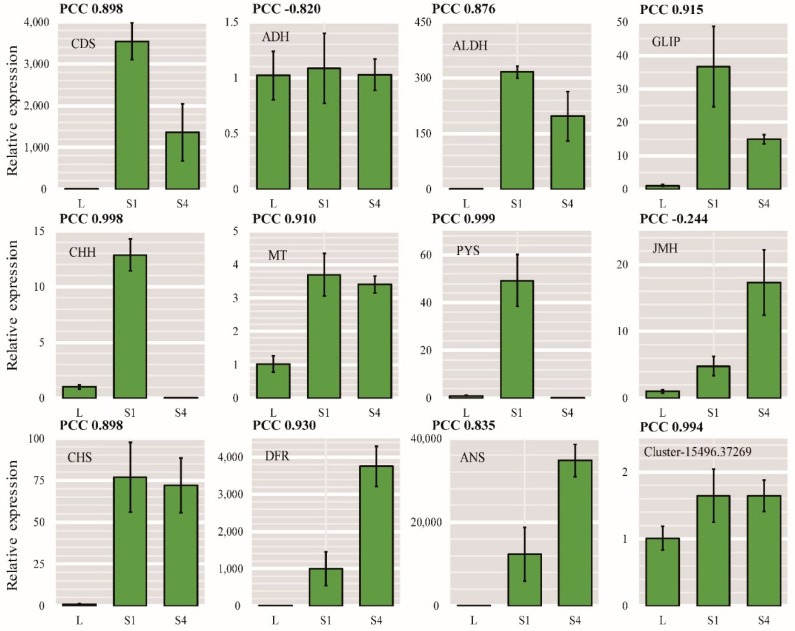
qRT-PCR validation of the genes related to the biosynthesis of pyrethrins and anthocyanin. Values are represented as averages ± standard deviations from three biological replicate samples. The PCC values (Pearson correlation coefficients) for the relative expression according to qRT-PCR compared with log_2_(FPKM+1) for RNA-Seq are indicated at the top of each graph.

To further verify the gene expression profiles obtained by RNA-Seq and the reliability of the important DEGs obtained from the assembled transcriptome, we selected eight key enzyme genes (i.e., CDS, ADH, ALDH, CHH MT, PYS, JMH, and GLIP) involved in pyrethrin biosynthetic pathways to verify their differential expression by qRT-PCR. Three of these genes were in the anthocyanin synthesis pathway (CHS, DFR, and ANS) and bHLH TF (Cluster-15496.37269). The results (Figure 6) show that the expression trends according to qRT-PCR were consistent with those indicated by the RNA-Seq data. Some differences may be due to the differences in the sensitivity, specificity, algorithms for qRT-PCR, or sequencing technique. The results obtained from the qRT-PCR analysis confirmed that the unigenes obtained from the assembled transcriptomes and the gene expression profiles from the RNA-Seq data were reliable.

### 2.9. Identification of Compounds

**Figure 7:**
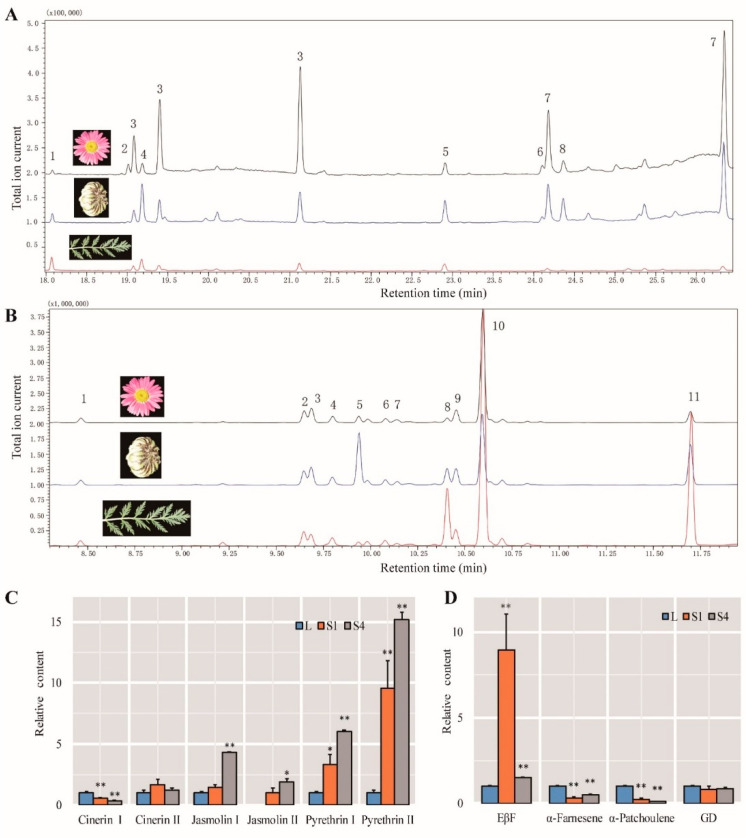
GC-MS analysis of compounds in leaves and flowering stages (S1 and S4). (**A**) Chromatogram profiles of *T. coccineum* leaves and flowering stages (S1 and S4) for extracts analyzed by GC-MS at the ion current (EIC) for m/z 123 and 107. 1. cinerin I; 2. jasmolin I; 3. pyrethrin I; 4. unknown 1; 5. cinerin II; 6. jasmolin II; 7. pyrethrin II; 8. unknown 2. (**B**) GC-MS analysis of terpene in leaves and flowering stages (S1 and S4) at the ion current (EIC) for m/z 93. 1. Elixene; 2. unknown; 3. caryophyllene I; 4. β-copaene 1; 5. (E)-β-famesene; 6. γ-muurolene; 7. linalool; 8. α-bergamotene; 9. germacrene D; 10. α-farnesene; 11. α-patchoulene. (**C**) The relative content of pyrethrins. The value for L was set as 1 and used as the reference, except for jasmolin II, as its content was too low to measure in the L stages, so the value of S1 stages was set as a reference. (**D**) The relative content of terpenes. The value for L was set as 1 and used as the reference. Error bars represent the standard deviations of the mean values of three replicates, and significant differences (*: *p* < 0.05; **: *p* < 0.01) were tested using Student’s paired *t*-test.

GCMS was used to quantify the contents of pyrethrins. The six components of pyrethrins could be detected in *T. coccineum* (Figure 7A), and the content of pyrethrins in the leaves was very low but increased in the S1 stage, after which it decreased in the S4 stage (Figure 7C). This is consistent with the observation of the content of pyrethrin in *T. cinerariifolium*. Pyrethrin synthesis genes were mainly expressed in the S1 stage, but pyrethrin was accumulated in the S4 stage. In addition, we also found two characteristic peaks of No.4 and No.8 closely following pyrethrins I and pyrethrins II, respectively. Their content was very high in the S1 stage but decreased in the S4 stage. Whether this may be from an intermediate substance in pyrethrin synthesis needs further research and observation. The mass spectrograms are shown in Supplementary Figure S4.

We also analyzed the proportions of six pyrethrins; the most predominant component was the I and II types of pyrethrins, followed by cinerins, and jasmolins showed the lowest content. Regarding the composition of the pyrethrins, there are differences between *T. coccineum* and *T. cinerariifolium*. Type I pyrethrins are more abundant than type II in *T. cinerariifolium*. However, the content of type II pyrethrins is higher than that of type pyrethrins I in *T. coccineum*.

Volatile terpenes play a vital role in *T. cinerariifolium*, such as defense, attracting insects, and pollination. In addition, these volatile terpenes and pyrethrins are synthesized from the same precursor pool. Therefore, the contents of the main volatile terpenes were measured. The main volatile terpenes were different in the L, S1, and S4 stages. Unlike EβF and GD, which were the dominant volatile terpenes in *T. cinerariifolium*, the main dominant VOC in *T. coccineum* was α-farnesene. The contents of EβF and GD in *T. coccineum* were low (Figure 7B). EβF was significantly up-regulated in the S1 stage, while the expression was lower in the L and S4 stages. The GD content was relatively constant, while for α-farnesene, its expression was the highest in the leaves, but it was significantly down-regulated during flowering (Figure 7D). In addition, both α-bergamotene and α-patchoulene were down-regulated as flowering progressed. The mass spectrograms are shown in Supplementary Figure S5.

### 2.10. Insect Test

**Figure 8:**
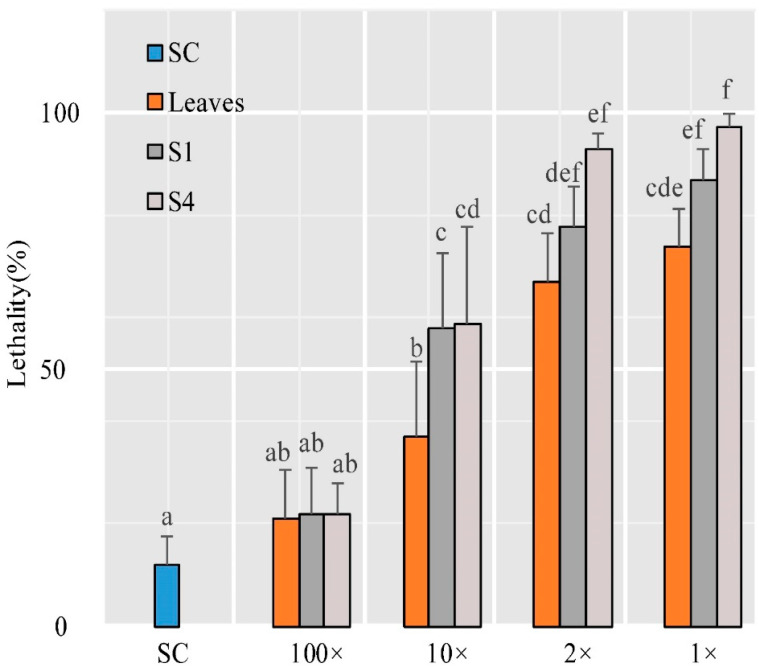
Insecticidal effect analysis of leaves and flowers in S1 and S4 stages. The ordinate represents the aphid lethality, and the abscissa represents the leaf disks treated with various concentrations of extract. The samples shown were diluted with 5% alcohol. 1×: undiluted; 2×: 2-fold dilution; 10×: 10-fold dilution; 100×: 100-fold dilution. Leaf disks treated with 5% alcohol were used as the solvent control. Error bars represent the standard deviations of the mean values of five replicates, and statistical analysis was performed by a one-way ANOVA test, followed by a post hoc Tukey test. The same letters indicate no significant difference; different letters (a–f) indicate significant differences (*p*-value < 0.05).

In order to determine the insecticidal effects of leaves and flowers in the S1 and S4 stages of *T. coccineum*, green peach aphids (Myzus persicae) were used for the aphid test. The results show that there was no significant difference in aphid mortality between the test samples and solvent control (leaf disk impregnation; 5% ethanol solution), while the extracts from pyrethrum leaves and flowers of S1 and S4 were diluted 100-fold. This suggests that there was almost no insecticidal effect on aphids under this extract solution. However, all the samples from leaves and flowers presented noticeable insecticidal activities when the extracts were diluted 10-fold. The extracts of flowers in the S1 and S4 stages showed higher activity than that of leaves. Furthermore, all of the extracts could kill more than half of the test aphids when they were diluted 2-fold. Comparing the extracts in the leaves and flowers in the S1 and S4 stages, the insecticidal efficacy seemed to be related to the content of total pyrethrins. Despite the total pyrethrin content differences, the extracts of flowers in the S1 and S4 stages did not show significant differences in mortality rates. This suggests that the content of pyrethrins is not the only element determining the insecticidal activity of *T. coccineum* (Figure 8).

## 3. Discussion

*T. coccineum* has significant ornamental value and displays large and colorful flowers that attract generalist pollinators. Moreover, it contains pyrethrins that have efficient insecticidal action. It is extensively utilized as an ornamental or intercropped plant in gardens and fields. Therefore, the high-throughput omics study of *T. coccineum* and research into its metabolites have substantial academic value and industrial significance.

Transcriptome sequencing is an effective tool for large-scale gene identification and expression profile analysis. In this study, the transcriptomes of three typical stages of *T. coccineum* were determined by high-throughput RNA-Seq. A total of 89,473 unigenes were obtained, and a large number of genes related to pyrethrin synthesis were found by homologous alignment and annotation. Many DEGs were also involved in the biosynthesis and metabolism of volatile terpenes and anthocyanins. This provides helpful information for further understanding the biosynthesis pathways for pyrethrins and other secondary metabolites in *T. coccineum*.

The biosynthesis of pyrethrins is a long and complicated process. In the present study, we analyzed the expression of pyrethrin biosynthesis genes at different stages. The results show that the pyrethrin synthesis genes in *T. coccineum* were extremely conserved compared to the same genes in *T. cinerariifolium*. The expression of pyrethrin biosynthesis genes showed a significant co-expression trend: low expression in leaves and upregulation in the S1 stage, followed by a decrease in the S4 stage, except for the constant ADH expression and JMH expression, which continued to rise. This shows that *T. coccineum* expresses many pyrethrin synthesis genes in the S1 stage more strongly. However, the content of pyrethrins was the highest at the S4 stage. These results show that *T. coccineum* has a period of pyrethrin synthesis similar to that in *T. cinerariifolium*. The production of pyrethrins may protect the most important seeds [33]. In addition, we analyzed the expression of anthocyanin biosynthesis genes at different stages. The results show that, while the early anthocyanin biosynthetic genes were expressed in S1, the late biosynthetic genes for anthocyanin were mainly expressed in the S4 stage. They produce the main flower colors of the full flowering stage that attract pollinators.

As derivatives of monoterpenoids, pyrethrins and other terpene compounds share a precursor pool. Isotopic labeling studies indicate that the acid moiety of pyrethrins is derived from the MEP pathway [34]. By contrast, volatile terpenes are mainly derived from the precursors of DMAPP and IPP synthesized by the MVA pathway [35]. The study in *Artemisia annua* showed that the synthesis of artemisinin competes with other terpene synthesis branches. Inhibiting other terpene synthesis branches can increase the synthesis of artemisinin [36]. In *T. cinerariifolium*, both EβF and GD accounted for almost 70% of the total terpenes in the bud stage [17,24]. Therefore, it is very conceivable that a considerable number of volatile terpenes exist in *T. coccineum*. Interestingly, the content of volatile terpenes was maintained at a low level in the full-bloom stage of *T. coccineum*. It is difficult to know whether or not the low concentrations of volatile terpenes play a role in plant defense. Therefore, *T. coccineum* seems to have lost the use of volatile terpenes as an indirect defense during its evolution. In insect tests, the insecticidal efficacy of the leaves and flower stages were significantly affected by the pyrethrin contents. However, despite the S1 and S4 flower stages presenting significant differences in their pyrethrin contents, they did not show significant differences in insecticidal efficacy. The contribution of volatile terpenes to the insecticidal activity of pyrethrins can almost be excluded under this extraction method. Other non-volatile substances in flower buds may enhance insecticidal activities. Overall, *T. cinerariifolium* can produce deadly, metabolically costly pyrethrins and high concentrations of volatile terpenes, while *T. coccineum* produces lower amounts of pyrethrins and low concentrations of volatile terpenes.

This seemingly paradoxical phenomenon is what triggered our interest. A possible explanation is that the pyrethrum is an insect-borne plant with insecticidal effects. It can kill insects via pyrethrins. Moreover, as a highly heterozygous self-sterile insect-borne flowering plant, it needs insects for pollination [21]. Reproduction is vital for the continuation of this species, so the pyrethrum must find a balance between the need for defense and the necessity of pollination. Although pyrethrum pollen contains almost no pyrethrins and is harmless to pollinating insects that only feed on pollen, most Lepidoptera and sawflies are both pollinators and herbivorous insects [37], to which the pyrethrins are likely to be undesirable or even fatal. In addition, though EβF defense against aphids can be observed under laboratory conditions, there are almost no aphids in the flowering pyrethrum [17]. The release of EβF as an aphid alarm hormone or as a pyrethroid synergist is redundant in plant defense. The release of defensive VOCs often requires large amounts of energy, so they are usually released when herbivorous plants are disturbed [38]. In *T. cinerariifolium*, these VOCs are almost constitutively released at the flowering stage [17]. Moreover, due to the inconspicuous flower form and color of *T. cinerariifolium*, the flower has difficulty attracting common pollinators. Therefore, the pyrethrum may use VOCs to attract potential pollinators, such as using EβF to attract hoverflies and lady beetles. The circadian rhythm of the release of GD may also help to attract night moths to achieve pollination. This may also explain the low VOC release from *T. coccineum*. Butterflies prefer bright and colorful flowers [39]. *T. coccineum* can maintain its attraction to pollinating insects through its larger flower diameter, contrasting red ray flowers, and yellow disc flowers, without the need for VOCs to attract insects. Therefore, although *T. coccineum* and *T. cinerariifolium* have similar patterns of pyrethrin synthesis, different strategies are adopted to regulate plant defense and plant pollination. These two species may be excellent materials for studying the strategies of balancing plant defense and pollination.

## 4. Materials and Methods

### 4.1. Plant and Insect Materials

Field-grown *T. coccineum* from the Flower Nursery Stock Base of Huazhong Agricultural University in Wuhan, China, was collected for the transcriptome analysis and GCMS analysis. The flesh leaves, flower buds (S1: well-developed closed buds), and full-blooming flowers (S4: three rows of disc florets were open) were harvested from biennial plants with appropriate conditions and consistency (the plant height was around 30 cm) and stored at −80 °C until use.

Green peach aphids (*M. persicae*) were collected from insecticide-free, lab-grown *Brassica napus* seedlings and reared on *B. campestris* in a climate room (16 h light photoperiod; 60 % relative humidity; 25 °C). Nymphs of approximately the same size were used for the experiments.

### 4.2. RNA Isolation, cDNA Library Construction, and RNA Sequencing

Total RNA was extracted using the RNeasy Plant Mini kit (Qiagen, Hilden, Germany). The quality and integrity of the total RNA were assessed using 1% agarose gel electrophoresis and the RNA Nano 6000 Assay Kit with the Agilent Bioanalyzer 2100 system (Agilent Technologies, Santa Clara, CA, USA). Sequencing libraries were generated using the NEBNext®Ultra™ RNA Library Prep Kit for Illumina® (NEB, Ipswich, MA, USA). The PCR products were purified (AMPure XP system Beckman Coulter, Brea, CA, USA), and the library quality was assessed on the Agilent Bioanalyzer 2100 system. The clustering of the index-coded samples was performed on a Bot Cluster Generation System using the TruSeq PE Cluster Kit (Illumina, San Diego, CA, USA), and the sequencing was performed by a commercial sequencing service provider (Novogene, Beijing, China).

### 4.3. De novo Assembly and Clustering

Raw data (raw reads) in the fastq format were firstly processed using the fastp software with the default parameters [40], and cleaned by removing adapter, poly-N, and low-quality reads. The Q20, Q30, GC-content, and sequence duplication levels of the clean data were then calculated. Transcriptome assembly was accomplished using Trinity v2.6.6 [41] with min kmer cov set to 2 and all the other parameters set to default. The quality of the transcriptomes was evaluated using N50 and BUSCO v3.0.2 [42].

### 4.4. Sequence Annotation and Functional Characterization

Gene function was annotated using the following databases: Nr (NCBI Non-Redundant Protein Sequences); Nt (NCBI Non-Redundant Nucleotide Sequences); Pfam (Protein Family); KOG/COG (Clusters of Orthologous Groups of Proteins); Swiss-Prot; KO (KEGG Ortholog Database); GO (Gene Ontology).

The clean reads were mapped to the unigenes with Bowtie2 v2.3.4 [43], and the expression levels of the genes were calculated using RSEM v1.3.1 [44] and normalized to fragments per kilobase per million (FPKM) values [45]. The principal component analysis (PCA) and correlation analysis of these samples were performed using OmicStudio tools (https://www.omicstudio.cn/tool, accessed on 18 July 2021).

### 4.5. Differential Expression Analysis

The differential expression analyses of two groups were performed using the DESeq2 v1.10.1 R package with the thresholds |log_2_FC| > 1 and Padj < 0.05 [46]. GO and KEGG enrichment analysis of the DEGs were performed using the R package ClusterProfiler [47]. The resulting *p*-values were adjusted using Benjamini and Hochberg’s approach for controlling the false-discovery rate.

### 4.6. Quantitative Real-Time PCR (qRT-PCR) Analysis

To validate the results of the RNA-Seq, DEGs related to the pyrethrin biosynthetic pathway genes, anthocyanin synthase genes, and transcription factors were chosen for qRT-PCR. The reverse transcription of the RNA sample was performed using a reverse transcription kit (Toyobo, Osaka, Japan). The specific primers were designed using the Primer Premier 5.0 software (Premier Biosoft, palo Alto, CA, USA) (the specific primers are shown in Supplementary Table S2) and checked using Tbtools [48]. qRT-PCR was run using the SYBR premix Ex Taq Kit (Takara, Kusatsu, Japan) with the Roche LightCycler® 96 Real-time PCR System (Roche, Basel, Switzerland). The qRT-PCR system with a total volume of 20 μL contained 10 μL of SYBR MIX, 0.4 μL of upstream primers, 0.4 μL of downstream primers, and 0.5 ng of cDNA template, and the amplification program was as follows: pre-denaturation at 94 °C for 30 s, followed by 40 cycles of denaturation at 94 °C for 5 s, renaturation at 60 °C for 15 s, and extension at 72 °C for 10 s. The relative expression levels were calculated using the 2^−ΔΔCT^ method with three biological and three technical replicates [49], with *Tanacetum GADPH* as the internal reference gene, and using Pearson’s correlation coefficient to measure the concordance with the expression profile of RNA-Seq.

### 4.7. Gas Chromatography/Mass Spectrometry Analysis

The samples were ground into powder using liquid nitrogen with mortars and pestles. Then, 0.5 g of powder was transferred into a tube containing 1 mL of MTBE with 0.01 ng/mL tetradecane as an internal standard. The tube was vortexed for 3 min at maximum speed, incubated at 24 °C with rotation at a speed of 50 rpm for 1 h, and then dried using Na_2_SO_4_ and filtered through a 0.22 mm-mesh filter.

GC/MS analysis was performed using GC/MS-QP2010Ultra apparatus (Shimadzu Corporation, Kyoto, Japan) with an HP-5 MS column. The column temperature was programmed as follows: holding at 40 °C for 3 min, 40 °C to 280 °C at 10 °C/min, and holding for 2 min. The ion source and transfer line temperatures were 230 °C and 280 °C, respectively; the electron ionization was 70 eV; and the mass scanning was performed from 45 to 450 m/z with 5 scans/s. The results were qualitatively and quantitatively analyzed using the GC Solutions software (Shimadzu Corporation, Kyoto, Japan) with NIST (2017) and the PESTEI3 library.

### 4.8. Insect Test

According to Li’s method [50], the leaves, buds (S1), and full-bloom (S4) flowers of *T. coccineum* were separately dried down in an oven at 50 °C for 48 h and ground into powders, and then, 20 g of powder was dissolved in 200 mL of n-hexane in glass bottles. Then, the samples were vortexed for 30 s, exposed to ultrasound for 10 min, and vortexed for another 30 s; then, they were centrifuged at 3000 revolutions per minute for 5 min. The supernatants were collected and evaporated. After the complete volatilization of the n-hexane, the supernatants were re-dissolved with 1 mL of absolute ethanol and then mixed with 19 mL of doubly distilled water to produce a 5% final concentration of ethanol.

The insect test was conducted through a leaf dip assay on agar beds as described by the Insecticide Resistance Action Committee (IRAC no.19 https://irac-online.org, accessed on 26 October 2021), with slight modifications. Flesh *B. campestris* leaf disks (30 mm diameter) were separately dipped in the test leaves and S1 and S4 extract solutions with a series of solutions diluted with 5% ethanol (1×: undiluted; 2×: 2-fold dilution; 10×: 10-fold dilution; 100×: 100-fold dilution) for 5 min with five replicates, and leaf disks dipped in 5% ethanol solution were the solvent control. After wiping them dry with filter paper, the leaf disks with the dorsal surface up were placed in 30 mL containers lined with solidified agar (10 mL of 7.5% agar) to maintain the leaf moisture. Each leaf disk was inoculated with 20 aphids of approximately the same size. The containers were sealed with lids with small air holes to prevent the aphids from escaping and kept in a climate room (16 h light photoperiod; 60 % relative humidity; 25 °C). The mortality of the aphids after 48 h of treatment was observed.

## 5. Conclusions

*T. coccineum* is a plant with great ornamental and pesticidal value. Previous research has mainly focused on the commercialized plant *T. cinerariifolium*, with higher pyrethrin contents. For *T. coccineum*, there are only a few records in public databases. Therefore, the present study focused on the biosynthesis pathways for pyrethrins, volatile terpenes, and anthocyanins. The results show that, compared to *T. cinerariifolium*, *T. coccineum* has lower amounts of pyrethrins and volatile terpenes but expresses late anthocyanin biosynthesis genes more strongly in the S4 stage, along with having large and colorful flowers. It seems likely that *T. coccineum* adopts a different strategy from *T. cinerariifolium* to balance plant defense and pollination. As the first report on the transcriptome of *T. coccineum*, this study can provide helpful genomic information for the further exploitation of and facilitate applications of *T. coccineum*.

## Data Availability

All the raw data can be downloaded from NCBI (BioProjects: PRJNA730666, https://www.ncbi.nlm.nih.gov/sra/PRJNA730666, accessed on 7 October 2021).

## Supplementary Materials

The following are available online at https://www.mdpi.com/article/10.3390/ijms222313005/s1.
