# Supplementary material for: Transcriptional Responses and GCMS Analysis for the Biosynthesis of Pyrethrins and Volatile Terpenes in Tanacetum coccineum
Source: Int J Mol Sci. 2021 Nov 30;22(23):13005. doi: 10.3390/ijms222313005 (PMC8657971; doi:10.3390/ijms222313005)
Supplement: Supplementary file 1 [file ijms-22-13005-s001.zip › ijms-1400178-supplementary-done.pdf]

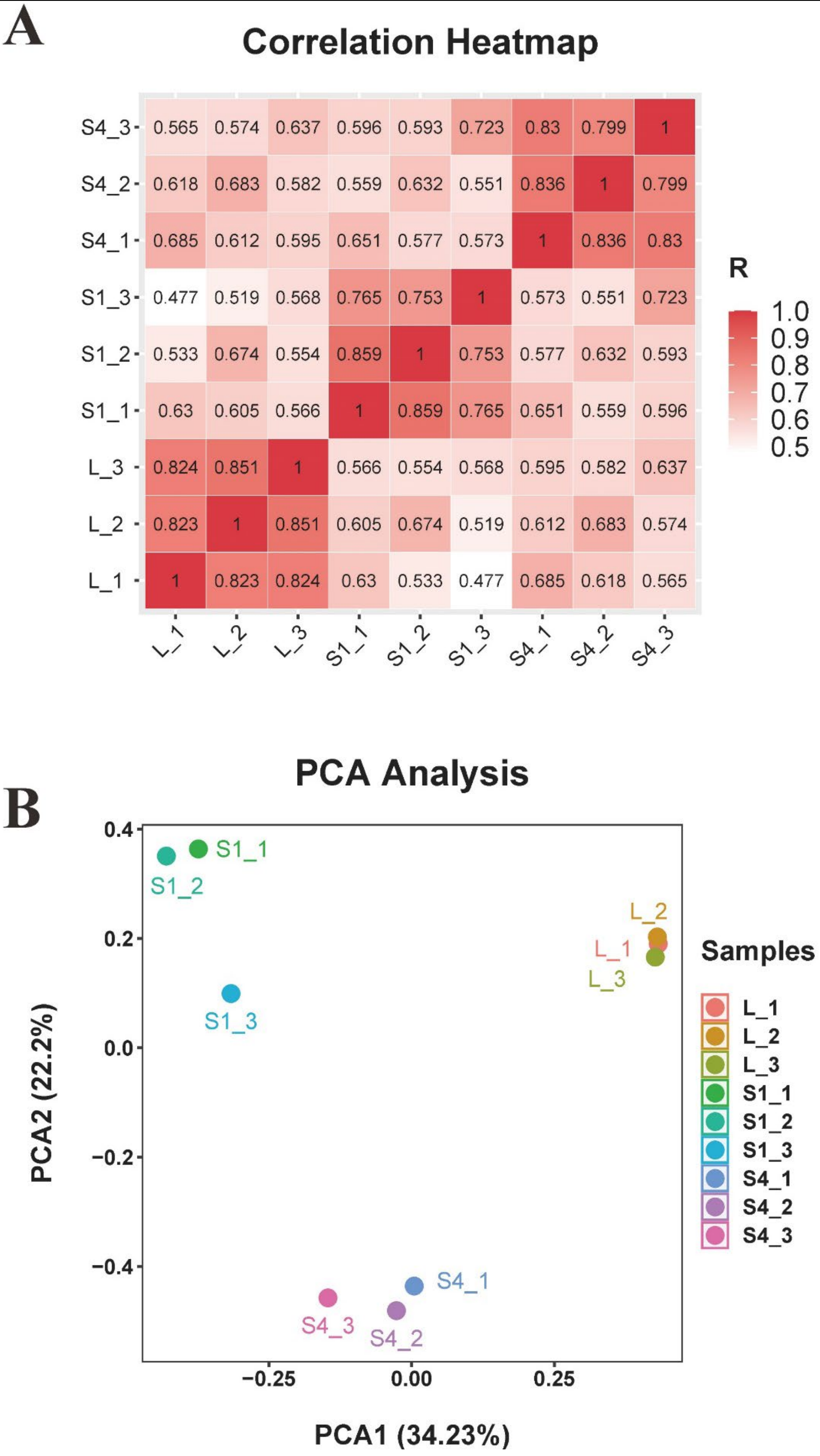

Figure S1 Correlation and PCA analysis of samples

A: Correlation analysis of samples; B: Principal Component Analysis of samples

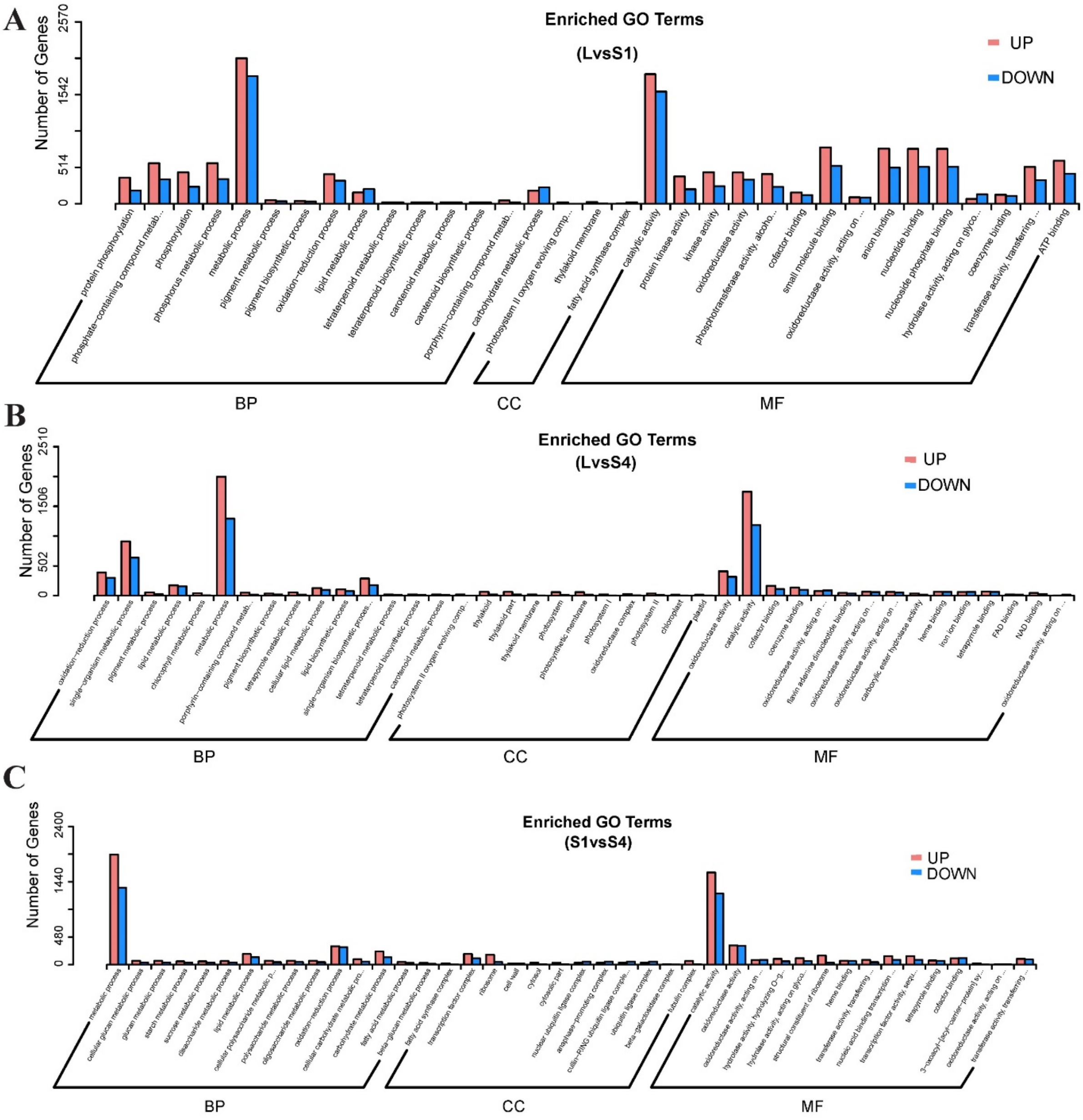

Figure S2. GO enrichment analysis of DEGs.

A: L vs S1; B: L vs S4; C: S1 vs S4

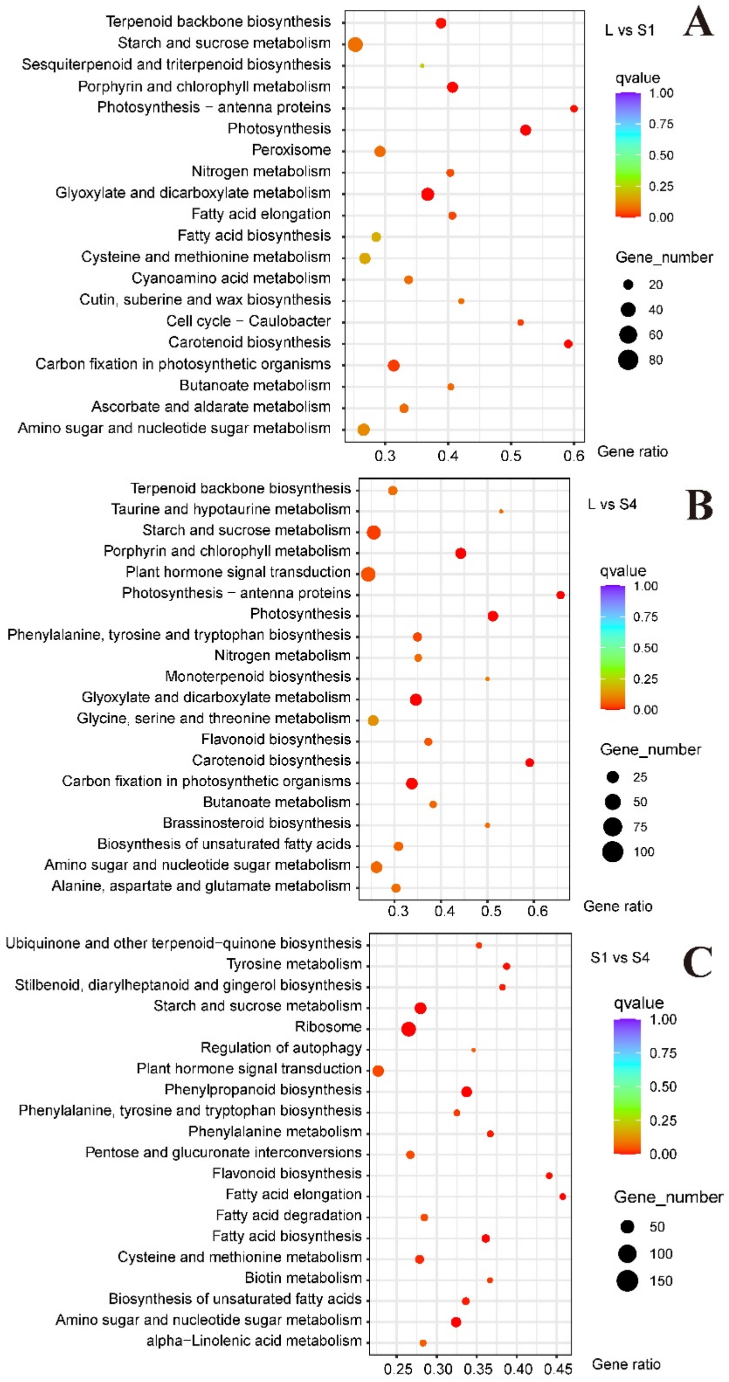

Figure S3 KEGG enrichment analysis of DEGs.

A: L vs S1; B: L vs S4; C: S1 vs S4.

Elixene C15H24 204

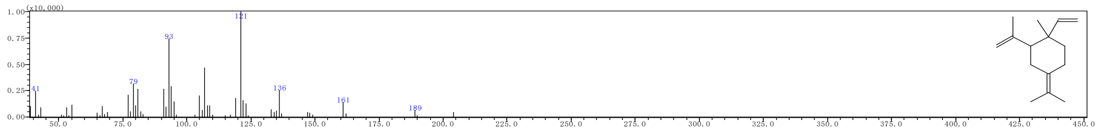

beta.-copaene C15H24 204

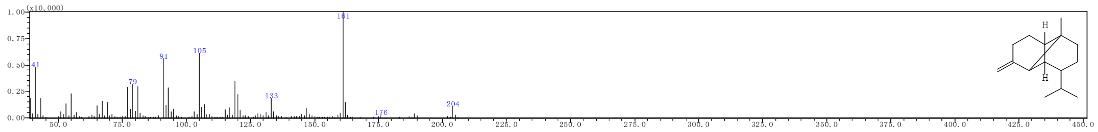

beta.-Caryophyllen C15H24 204

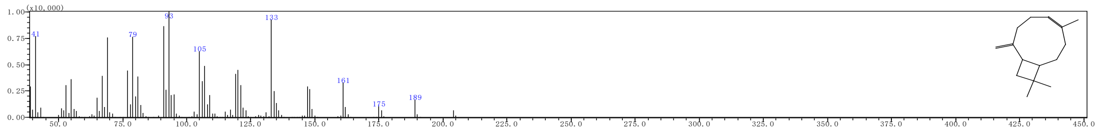

(E)-.beta.-Famesene C15H24 204

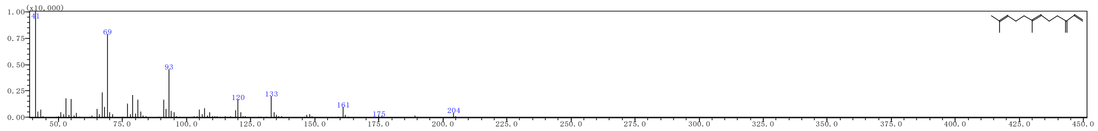

alpha.-Bergamotene C15H24 204

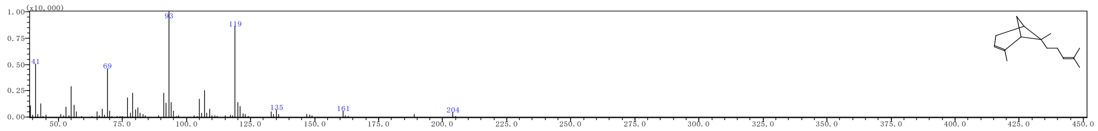

Germacrene D C15H24 204

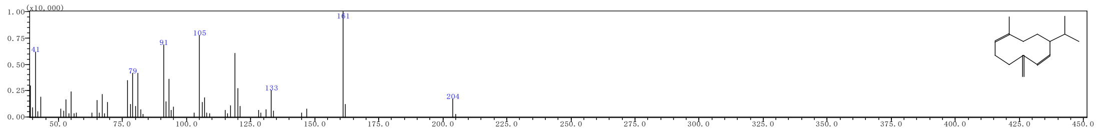

alpha.-Farnesene C15H24 204

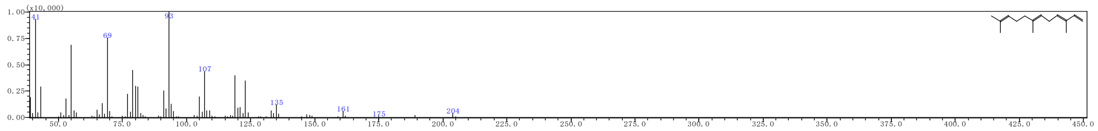

Figure S4 The mass spectrograms of pyrethrins

Cinerin I C20H28O3 316

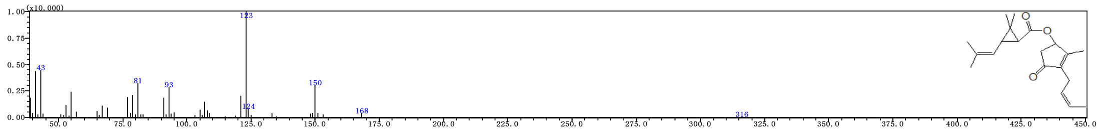

Jasmolin I C21H30O3 330

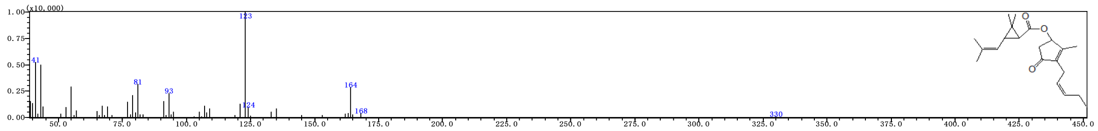

Pyrethrin I C21H28O3 328

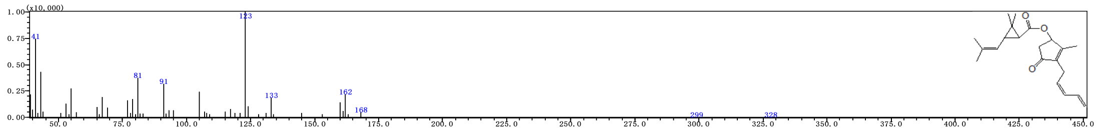

Unknown 1

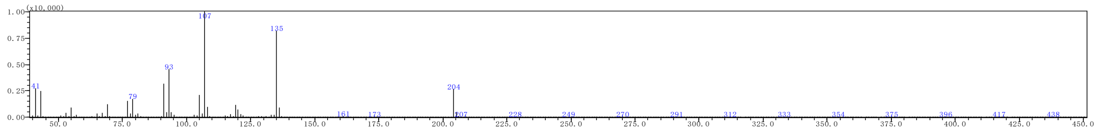

Cinerin II C21H28O5 360

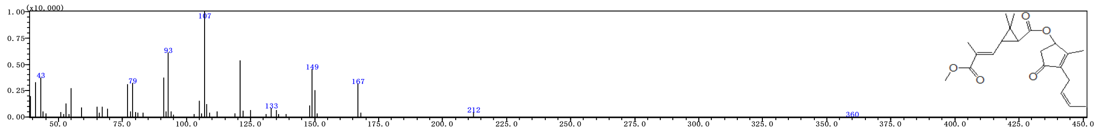

Jasmolin II C22H30O5 374

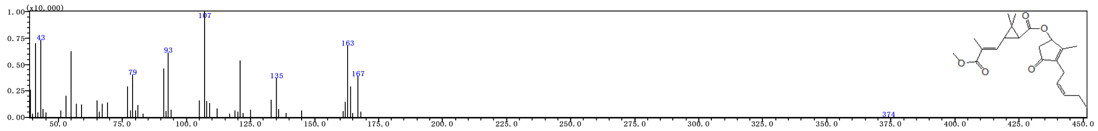

Pyrethrin II C22H30O5 374

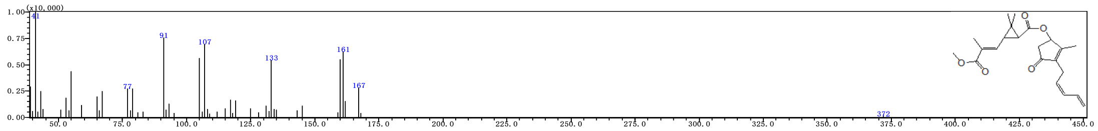

Unknown 2

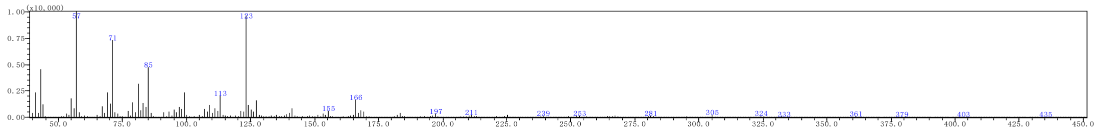

Figure S5 The mass spectrograms of volatile terpenes

Table S1. Summary of Illumina HiSeq™ 4000 sequencing data

Sample	Raw reads	Clean reads	Clean bases(bp)	Error(%)	Q20(%)	Q30(%)	GC content(%)
L_1	51087630	48713978(95.35%)	7.31G	0.03	97.95	93.77	43.05
L_2	44783836	43123200(96.29%)	6.47G	0.03	97.88	93.59	43.38
L_3	45877880	43562156(94.95%)	6.53G	0.03	97.68	93.15	43.13
S1_1	47513258	45032560(94.78%)	6.75G	0.03	97.76	93.31	42.31
S1_2	46556522	45149940(96.98%)	6.77G	0.03	97.91	93.65	42.29
S1_3	43845892	42370462(96.63%)	6.36G	0.03	97.72	93.23	42.09
S4_1	45180438	44016074(97.42%)	6.6G	0.03	97.84	93.51	42.37
S4_2	43971966	42554940(96.78%)	6.38G	0.03	97.88	93.54	42.37
S4_3	46138630	43929062(95.21%)	6.59G	0.03	97.97	93.75	41.99

Q20: Percentage of bases that account for more than 20 of Phred values. Phred=-10log10(e).

Q30: Percentage of bases that account for more than 30 of Phred values.Phred=-10log10(e).

GC content: The sum of base G and C accounts for the percentage of total base number.

Table S2. qRT-PCR primer

Primer	Primer sequence (5'to3')
DFR_RT_F	ATGCCACCATTCATCAATTAGC
DFR_RT_R	CCTCCGAACATCTCCTCCAA
CHS_RT_F	GGCTGATTACCAACTCACCAA
CHS_RT_R	TCGTTAGGACCACGGAATGT
ANS_RT_F	TCCAACTCTTCTACGACAACCA
ANS_RT_R	TCCTAACCTTCTCCTTATTCACAAG
GLIP_RT_F	CGATGAACTTACTAGACTACACAA
GLIP_RT_R	AAGGACCACTACCGCAACAT
ADH_RT_F	GGTGAATGTGGACAATGCT
ADH_RT_R	CGGACCATGTGGAGCAACT
CDS_RT_F	TTCTCCTCTCACTACGACATTGA
CDS_RT_R	ACAGAATAGCCTCGGACCATC
JMH_RT_F	TTTGTTGCGGATCTTTGGC
JMH_RT_R	GTCTTCCTGATCATGAGAATTGG
MT_RT_F	TTCATTCCTCAACCAGCCTACA
MT_RT_R	ACTATTTCGATAGAGCGTGATT
bHLH1_RT_F	TTCTGATACCACGGCTACTA
bHLH1_RT_R	GCCAGACAATCCTTGATGAT
CHH_RT_F	CCACGTGTATGTAGGGAGCAATGC
CHH_RT_R	CAGGATCAGTTGAACATGCGAAAG
ALDH_RT_F	CATTCCGCTACTTTGCTGGTGC
ALDH_RT_R	TCCAAGGAATGATGTGTCCAACTAC
PYS_RT_F	CTACAAGGTGCCCCCAAAGA
PYS_RT_R	TGATGACGGCTCCAAGGTAGA
